# Design Modifications in Distal Shoe Therapy: A Scoping Review

**DOI:** 10.7759/cureus.88672

**Published:** 2025-07-24

**Authors:** Ritu J Sabharwal, Kanika Singh Dhull, Brahmananda Dutta, Anandamoy Bagchi, Sudipta Kar, Adrish Misra

**Affiliations:** 1 Pediatric Dentistry, Kalinga Institute of Dental Sciences, Bhubaneswar, IND; 2 Pedodontics and Preventive Dentistry, Kalinga Institute of Dental Sciences, Bhubaneswar, IND; 3 Pediatrics and Preventive Dentistry, Kalinga Institute of Dental Sciences, Bhubaneswar, IND; 4 Pediatric Dentistry, Guru Nanak Institute of Dental Sciences and Research, Kolkata, IND; 5 Dentistry, Kalinga Institute of Dental Sciences, Bhubaneswar, IND

**Keywords:** distal shoe, evidence-based dentistry, mixed dentition, space maintainers, tooth eruption

## Abstract

This review aims to present updated evidence on different modifications of distal shoe space maintainers, which will aid in optimizing patient outcomes and standardizing procedures. Distal shoe space maintainer therapy is needed to prevent space loss and guide the eruption of the permanent first molar when the primary second molar is lost prematurely before the eruption of the permanent first molar. Studies assessing the efficacy of different distal shoe space maintainer adjustments in children with mixed dentition were found through a thorough search of electronic databases. Data extraction and screening were carried out by two separate reviewers. Nine case reports evaluating nine children in the mixed dentition phase, ages three to six, were included in the qualitative synthesis. In cases of bilateral primary molar loss, the distal shoe space maintainer, an effective tool for both preventive and interceptive treatment, is a reliable way to keep space. This review will provide clinicians with an essential, evidence-based resource to optimize distal shoe therapy, leading to better long-term oral health for children. In pediatric dentistry, ensuring effective treatment with minimal discomfort is essential for patient cooperation and efficient care. The distal shoe space maintainer supports this goal by guiding proper eruption of permanent teeth, especially after premature loss of primary molars. It plays a key role in preserving arch length and preventing future malocclusion, aligning with preventive and interceptive orthodontic principles.

## Introduction and background

One of the primary challenges in pediatric dentistry is preserving primary teeth in the dental arch until their natural exfoliation occurs [1]. However, premature tooth loss due to extensive caries or trauma is common [2]. Approximately 51% of premature primary first molar loss and 70% of premature primary second molar loss lead to space loss and misalignment of permanent teeth [3]. Premature loss of primary second molars and the lack of eruption guidance for permanent teeth can lead to a substantial decrease in arch length, exceeding 8 mm in each maxillary quadrant and 4-6 mm in each mandibular quadrant [4]. Space maintainers are necessary to stop space loss and the ensuing malocclusion [5].

The patient's age, occlusion, compliance, dental development stage, dental arch, and missing teeth all play a role in the choice of space maintainer. A review of the various modifications was necessary due to the limitations of the conventional appliance, including its unsuitability for medically compromised patients, interference with socket epithelialization, and potential to cause infection [6]. Premature loss of deciduous second molars poses a particular challenge. Theories about how to guide the eruption of permanent first molars are used to choose the best-suited space maintainer [7]. The first theory suggests that the distal crown surface of the primary second molar functions as a guide, necessitating a distal shoe design that extends into the tissue [8]. According to the second theory, a distal vertical blade on the mesial surface of the unerupted molar is necessary for the permanent first molar to erupt under the guidance of the main second molar's distal root [9].

In cases where the deciduous second molar is lost prematurely, i.e., before the eruption of the first permanent molar, a specialized intra-alveolar space maintainer is required [8]. The eruption paths of both the maxillary as well as mandibular first permanent molars differ such that the former erupt distally and buccally, whereas the latter erupt mesially and slightly lingually. These differences necessitate careful design and placement of space maintainers [9].

Gerber was the first to introduce the distal shoe appliance, which helps the first permanent molar to emerge properly [10]. It is indicated for early primary second molar loss, very extensive root resorption, bone loss in the periapical area, non-restorable primary molars, ectopic eruption of permanent molars, and ankylosed deciduous second molars [11]. The absence of a permanent first molar, poor abutments from serial tooth loss, lack of participation from the child or parents, and systemic disorders that interfere with wound healing, like diabetes mellitus and heart abnormalities that necessitate antibiotic prophylaxis, are contraindications [7,12].

In such cases, a pedodontist has the option to use either a non-invasive fixed or removable appliance that exerts pressure on the ridge mesial to the unerupted permanent molar, or to wait for the eruption of the first permanent molar before attempting space regaining. A scoping review was conducted to provide a comprehensive update on the modifications made to distal shoe space maintainers [12,13].

## Review

Eligible studies were selected based on inclusion criteria that required them to be published in English between January 2000 and December 2024 in open-access journals, involve children under six years or in mixed dentition, and include sufficient data on the effects of various distal shoe modifications, including case reports and case series. All the included studies are case reports.

The exclusion criteria included review articles, abstracts, letters to the editor, editorials, animal studies, in vitro studies, and articles published in journals with restricted access.

Children with mixed dentition under six years of age with premature loss of deciduous second molar, with exposure to the distal shoe appliance, preventing the deflection of the permanent first molar eruption path, were included.

A database search was conducted until December 2024, covering the past 24 years, using PubMed, Scopus, Google Scholar, and EBSCOhost. Boolean operators were used in conjunction with keywords and Medical Subject Headings (MeSH) terms: "tooth eruption" OR "primary dentition" OR "paediatric dentistry" OR "space maintainers" OR "space management" AND "distal shoe" OR "premature loss" AND "permanent first molar" AND "deciduous molar" AND "mixed dentition".

Screening and data extraction were carried out as follows: a two-phase screening was conducted by two independent reviewers. Initially, titles and abstracts were assessed, and irrelevant studies were excluded. Full-text articles were then reviewed, with discrepancies resolved through discussion. A third reviewer was involved in cases of disagreement. Data extraction was performed under the following categories: author(s), country, year, study population, distal shoe used, and appliance type.

**Figure 1:**
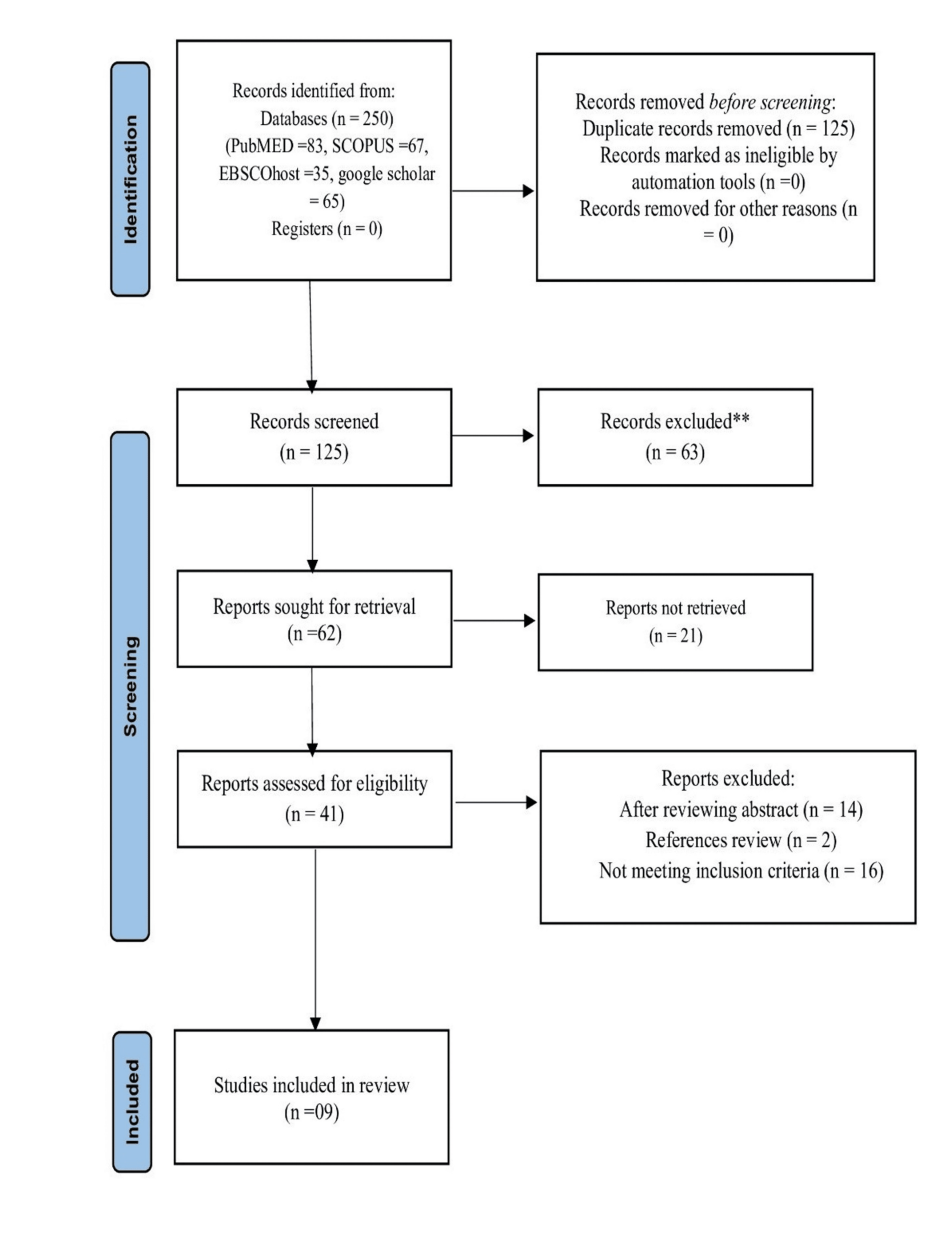
Flow diagram of study selection.

Of the 250 studies initially retrieved, nine met the inclusion criteria for qualitative synthesis, as illustrated in Figure 1.

Study characteristics are shown in Table 1, and accordingly, the data were evaluated from nine studies [13-21], including nine children in the mixed dentition period with ages ranging from three years to six years. All the included studies had a case report design. Among the included studies, five studies [13,14,16,17,21] were conducted in India, two studies [18,19] in Iran, and one study each in Libya [15] and Thailand [20]. Various distal shoe space appliance modifications have been described.

**Table 1:** Descriptive study details of all the included studies. DSSM: distal shoe space maintainer; SSC: stainless steel crown.

Author, year of study	Country	Study population	Distal shoe used	Appliance, follow-up/success rate
Dhindsa et al. (2008) [13]	India	A 4-year and 10-month-old girl presented with carious deciduous mandibular molars on both sides.	Willet appliance	A modified bilateral Willet’s appliance was fabricated with a short-term posterior design, while the anterior wire component resembled a lingual holding arch. At the 10-month follow-up, the lower right first and second premolars had erupted. The lower right permanent first molar also showed a pre-eruptive bulge, indicating imminent eruption. Based on this observation, the decision was made to remove the modified distal shoe appliance. One year later, the lower right permanent first molar successfully erupted into the oral cavity.
Dhull et al. (2011) [14]	India	A 5-year-old girl presented with a carious primary mandibular second molar.	Willet appliance, or intra-alveolar eruption guidance appliance	A modified Mink reverse band and loop appliance with an intra-alveolar extension was used. Follow-up appointments were scheduled every two months to monitor the condition of the appliance. After 1.5 years, the mandibular permanent first molar had erupted.
Gujjar et al. (2012) [15]	Libya	A five-year-old boy presented with a missing primary mandibular left first molar and a decayed primary mandibular left second molar.	DSA with stainless steel crown retainer	A lingual arch was extended bilaterally into a distal shoe using 0.8-mm orthodontic stainless steel wire. The appliance was utilized for a short duration of approximately two months.
Kiran DP et al. (2015) [16]	India	A 3-year-old boy presented with a decayed primary mandibular left second molar.	DSSM with stainless steel crown	A Willet appliance was used, and periodic recall visits were scheduled at three-month intervals. At the 12-month follow-up, the permanent first molar had erupted in alignment with the arm of the distal shoe, effectively simulating the surface of the missing deciduous second molar.
Somwanshi et al. (2016) [17]	India	A 4.5-year-old boy presented with the mandibular first and second primary molars on the right side and the mandibular second primary molar on the left side.	Willet appliance	A distal shoe space maintainer (DSSM) was used in conjunction with a stainless steel crown. At the 10-month follow-up, the permanent first molars had erupted, and the appliance was well tolerated by the child. It was subsequently planned to replace the DSSM with a bilateral fixed space maintainer, such as a lingual arch, once the permanent first molars and incisors had fully erupted.
Afshar et al. (2017) [18]	Iran	A 4.5-year-old boy presented with carious deciduous mandibular molars on both sides and bilateral loss of primary molars.	Gerber distal shoe appliance	A lingual arch wire was used. Six weeks after appliance placement, the permanent left first molar had erupted, while the permanent right first molar was in the process of erupting. After one year, once both first permanent molars had fully erupted, the distal shoe was removed, and the space from the distal surface of the canine to the mesial surface of the first molar was measured on both sides.
Mazhari et al. (2022) [19]	Iran	The molar relationship in a six-year-old child was identified as class II on the right side and class I on the left side.	Distal guide with SSC	A band and loop appliance was used, and an average follow-up period of six months revealed that the permanent tooth had erupted along its natural path.
Sakulratchata et al. (2022) [20]	Thailand	A 6-year-old boy presented with carious second primary molars on both the left and right sides of the mandible.	DSSM with SSC	A lingual arch was extended bilaterally. After one year and two months, with the complete eruption of the mandibular first permanent molars, the distal shoe appliance was removed and replaced with a lingual arch space maintainer.
Gupta et al. (2024) [21]	India	A 4-year-old boy presented with a carious mandibular primary first molar.	DSSM	A horizontal band and loop appliance was used. At the 24-month follow-up, the permanent mandibular first molar (tooth 46) was clinically visible. However, since tooth 46 had only partially erupted, it was decided to replace the horizontally looped distal shoe with a reverse band and loop space maintainer.

Discussion

For all the included studies, clinical success was consistently defined as the proper guidance of the erupting permanent first molar into its normal anatomical position. Previous studies have defined the criterion for assessing the success of a distal shoe space maintainer (DSSM) as the eruption of the unerupted permanent tooth into the dental arch without any appliance-related issues [7,8]. A clinically meaningful criterion of success is thus eliminated (i.e., Did the appliance accomplish its intended purpose for the patient while requiring maintenance or replacement during treatment?) [9]. The final goal of therapy should be considered as a success indicator because a child's connection with a pediatric dentist is dynamic rather than static [10].

A systematic evaluation was conducted in 2016 by El-Motayam et al. [22] to evaluate the effectiveness and safety of the DSSM. We searched through the databases to find research assessing space maintainers' efficacy and reporting gingival response and patient tolerance to DSSM. The review comprised six studies. Although it was shown that patients tolerated DSSM well, its effectiveness and gingival response could not be examined because of the lack of data and significant heterogeneity.

In 2022, Khalaf et al. [23] carried out a comprehensive evaluation to evaluate the efficiency of space regainers and maintainers in preventing as well as correcting the disparity in dental arches in mixed dentition. Database searches turned up cohort and case control studies, controlled clinical trials (CCTs), randomized controlled trials (RCTs), and class I, II, and III skeletal malocclusions that needed space maintainers or space regainers, as well as children with mixed dentition who had moderate to severe crowding. The final review comprised eleven trials that dramatically enhanced arch length using two space regainers (lip bumper and transpalatal arch) and nine space maintainers (lower lingual arch). It was found that space maintainers and regainers are very effective in reducing crowding and preserving arch length in children with mixed dentition.

A systematic evaluation was conducted by Dadpe et al. (2023) [24] to evaluate how well the DSSM prevents loss of space in the dental arch. Studies assessing the efficacy of DSSM in terms of gingival response and patient tolerance were found by searching databases. Eleven studies were reviewed. It was discovered that patients who had the most comfort cooperation and the least amount of chair-side time benefited the most from DSSM; however, the effect of DSSM on gingival response could not be explained because of the lack of adequate information.

For the assessment of the survival rate of space maintainers of any type, such as removable, fixed with a metal basis, or fixed with a resin base, in pediatric patients who have early primary tooth loss, Casaña-Ruiz et al. (2025) [25] carried out a meta-analysis and systematic review. Eleven studies were found when databases were searched for observational studies as well as RCTs. Because of their longer stability, lower risk of loss, and capacity to stop teeth movement, it is advised to use fixed space maintainers like lingual arch (LA) or band and loop (BL). Along with speech development, they are also crucial for masticatory function.

Few systematic reviews and meta-analyses [23-25] have addressed the topic, but none have provided a comprehensive qualitative analysis of the various modifications to DSSMs due to the heterogeneity of available data. To the best of our knowledge, this is the first scoping review to offer a detailed explanation of the different types of DSSMs.

The scoping review was conducted following the Preferred Reporting Items for Systematic Reviews and Meta-Analyses extension for Scoping Reviews (PRISMA-ScR) guidelines [26]. Scoping reviews are considered one of the most valuable forms of evidence, as they tackle specific research questions in a transparent and reproducible way. However, the strength of the evidence depends on the quality of the studies included. In this review, a sufficient number of studies with short observation periods and established methodological quality were available.

For all the included studies, clinical success was consistently defined as the proper guidance of the erupting permanent first molar into its normal anatomical position.

The clinical significance of this review lies in highlighting the role of distal shoe therapy in preserving space following premature loss of primary molars, thereby preventing drift of permanent molars and reducing the risk of future malocclusion. It explores technique variations and possible complications, consolidating current evidence to support the development of standardized protocols for appropriate appliance selection, placement, and maintenance. By offering evidence-based guidelines, the review aims to help clinicians reduce risks and enhance long-term oral health outcomes in children, ensuring proper development of the permanent dentition.

## Conclusions

In pediatric dentistry, one of the foremost goals is to deliver maximum therapeutic benefit to young patients while ensuring minimal discomfort during procedures. Achieving this balance not only enhances the quality of care but also improves the overall dental experience for children. Minimizing discomfort leads to better patient cooperation, which is critical in managing young, often anxious patients. Reduced chair time is another key aspect, as it contributes to patient comfort and allows for efficient clinical workflow. Within this context, the distal shoe space maintainer has been recognized as a useful tool in pediatric practice, particularly when applied in appropriately selected cases. It is especially valuable in preventive and interceptive orthodontic therapy, helping to guide the proper eruption of permanent teeth. This appliance is particularly beneficial in cases where there is premature loss of primary molars, a common issue in pediatric dentistry. In situations involving bilateral loss of primary molars, the distal shoe becomes indispensable for maintaining space and preventing undesirable tooth movement. Maintaining proper arch length helps avoid future malocclusion and complex orthodontic treatment. Thus, the use of distal shoe space maintainers aligns well with the primary objectives of pediatric dentistry: preserving oral health with minimal intervention and maximum cooperation.
